# The impact of Fe atom on the spin-filter and spin thermoelectric properties of Au-Fe@C_20_-Au monomer and dimer systems

**DOI:** 10.1038/s41598-020-78111-w

**Published:** 2020-12-03

**Authors:** H. Khalatbari, S. Izadi Vishkayi, H. Rahimpour Soleimani

**Affiliations:** 1 Computational Nanophysics Laboratory (CNL), Department of Physics, University of Guilan, Po Box: 41335-1914, Rasht, Iran; 2 School of Physics, Institute for Research in Fundamental Science (IPM), P. O. Box 19395-5531, Tehran, Iran

**Keywords:** Nanoscience and technology, Physics

## Abstract

Based on density functional theory and non-equilibrium Green’s function formalism, we explore the effect of Fe atom in Au-Fe@C_20_-Au monomer and dimer systems in comparison with the C_20_ fullerene molecular junctions. We calculate the spin-dependent transmission coefficient, spin polarization and also their spin thermoelectric coefficients to investigate magnetic properties in the system. Our results indicate that the presence of Fe atoms enhances substantially the spin-filter and increases the spin figure of merit in the dimer system. We suggest that the Au-(Fe@C_20_)_2_-Au system is a suitable junction for designing spin-filtering and spin thermoelectric devices and eventually it is a good candidate for spintronic applications.

## Introduction

C_20_ fullerene with I_h_ symmetry group is the smallest carbon cage with 12 pentagons. It breaks the isolated pentagon rule^1^. Synthesis of this fullerene by using C_20_H_20_ had been carried out in 2000^2^. C_20_ fullerene as one of the derivatives of C_60_ has a long lifetime at room temperature^3^. When two C_20_ fullerenes are approximately connected form a (C_20_)_2_ dimer. Fullerene dimers have wide consideration, due to their unique structures and properties^4–6^.

Endohedral metallofullerenes (EMFs), which have one or more metal atoms inside the fullerene cage, are novel forms of fullerene-based materials which have attracted wide interest, not only in physics and chemistry but also in such interdisciplinary areas as biological sciences and have been investigated for both main group and transition metals (TMs). The placement of TM atoms inside the C_20_ fullerene can be considered as a magnetically active center^7^. In fact, C_20_ is capable of protecting ferromagnetic materials from oxidation and also can reducing the magnetic interactions between them^8^. EMFs have been studied extensively due to their unique properties. For example, Li et al. have performed the magnetic properties of Ni@C_60_ by the spin-polarized density functional calculations^9^. Endohedral (TM@C_20_) and exohedral (TM-C_20_) (TM = Group 11 and 12 transition metal atoms/ions) were studied by Gonzalez et al.^10^. Also, Zhao et al. have investigated the stability, electronic and magnetic properties of the transition metals encapsulated C_20_ cage^11^. In addition, numerous other works have been done in this field^12–18^. In EMFs, the “@” symbol is used to represent that atoms at the left are encapsulated within the fullerene cage on the right.

The endohedral transition-metal-fullerenes have many interesting physical properties in the molecular spintronic devices, such as spin-filtering. The main goal of spintronics is to acquire the knowledge of spin-dependent phenomena and to apply them for more and modern applications. There are many works which have been done in this field, for example; Wu et al. had investigated the spin-polarized transport properties of Au-(Fe@C_60_)_2_-Au system in two parallel (P) and anti-parallel (AP) configurations by applying non-equilibrium Green’s function (NEGF) formalism combined with the density functional theory (DFT)^19^. Saffarzadeh et al. had studied the spin-dependent transport through Au-(Co@C_60_)_2_-Au system using DFT and extended Hückel theory^20^. Also they had calculated the spin polarization (SP) as a function of gate voltage. A theoretical study on the adsorption and the spin transport properties of Fe@C_28_ was reported by Xu et al.^21^ using spin DFT and NEGF techniques. Caliskan had investigated the SP of partial density of states (PDOS) and current–voltage characteristics of N@C_20_^22^ and X@C_70_ (X = B, N)^23^ attached to Au electrodes. In addition to the EMFs, the TM atoms, encapsulated in the other buckyballs molecule such as B_40_, can induce SP^24^. The placement of TM atoms has been reported also in numerous other structures^25–32^.

Thermoelectric properties of EMFs have been considered experimentally and theoretically in recent years. See Kei et al. have investigated the thermoelectric properties of molecular junctions based on C_82_ and its two EMF derivatives, Gd@C_82_ and Ce@C_82_, connected to Au electrodes^33^. Rincón-García et al. have studied single-molecule junctions of the Sc_3_N@C_80_ connected to Au electrodes by using a scanning tunnelling microscope^34^. Also, experimental observation of spin Seebeck effect was reported in several works^35,36^.

Despite all the attempts for finding the characteristics of EMFs, the spin-filtering and spin thermoelectric properties of TM@C_20_ have not reported yet. According to Ref.^22^, N@C_20_ attached to Au electrodes had spin-dependent current–voltage characteristics, therefore we are motivated to use C_20_ as the molecular bridge between Au electrodes. On the other hand, in Ref.^37^, it was observed that the thermopower and the figure of merit had increased with the number of fullerene molecules, so we have compared the transport properties of monomer and dimer C_20_. We explored all TM@C_20_ molecules and finally found that the magnetism pattern is maximized on the molecule which is doped by Fe in the middle. So, in this paper, we study the behavior of monomer and dimer Fe@C_20_ fullerenes between the Au electrodes. Here, we use NEGF formalism in combination with DFT to calculate the spin-dependent electrical and thermoelectric properties of the considered molecules. In fact, the main goal of the present study is to explore the potential of molecular junctions formed by monomer or dimer fullerenes for thermoelectric efficiency and spin-filter efficiency.

## Calculation methods and simulation model

**Figure 1:**
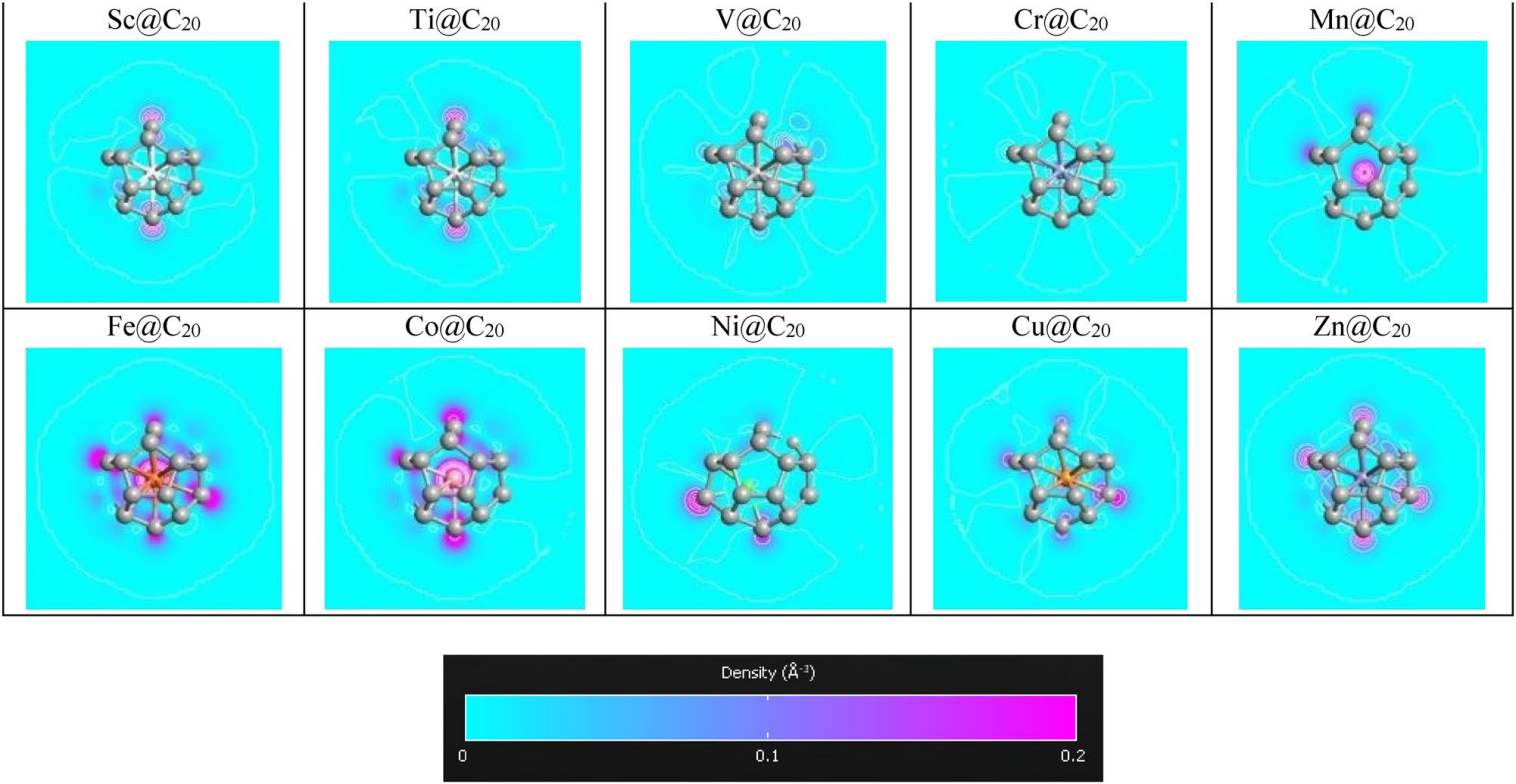
The electron density of TM@C_20_ molecules.

The isolated molecules (C_20_ and Fe@C_20_ monomer and dimer) are initially optimized by the spin polarized DFT calculations using SIESTA package^38^. The Perdew–Burke–Ernzerhof (PBE) parameterization of generalized gradient approximation (GGA) functional is applied for the exchange–correlation potential^39^ and the kinetic energy cutoff plane-waves is equal to 100 Hartrees. It is observed that TMs prefer to place at the center of C_20_^40^. The difference between spin-up and spin-downs Mulliken populations, the energy differences between the spin-polarized and non-spin-polarized (∆E) calculations and the binding energy (E_b_) of TM@C_20_ are listed in Table 1 and the electron density diagram of TM@C_20_ is drawn in Fig. 1. ∆E and Mulliken population of TM@C_20_ has a maximum value for TM = Fe or Co. On the other hand, the E_b_ of Fe@C_20_ is more than Co@C_20_, so we have focused on Fe@C_20_ molecule which is more favorable to form and also it has a desirable magnetism pattern.

**Table 1:** The differences between spin-up and spin-down Mulliken populations, the energy differences between the spin-polarized and non-spin-polarized (∆E) and the binding energy (E_b_) of TM atoms in C_20_ fullerene.

Atom	Sc	Ti	V	Cr	Mn	Fe	Co	Ni	Cu	Zn
Mulliken	0.18	0.17	0.07	0	0.82	3.32	2.44	0.08	0.09	0.05
∆E (meV)	124.47	35.12	6.9	0	97.18	1649.43	1940.99	103.21	290.58	214.99
E_b_ (eV)	− 6.30	− 6.47	− 6.46	− 6.28	− 6.21	− 6.15	− 6.09	− 6.15	− 5.91	− 5.80

**Figure 2:**
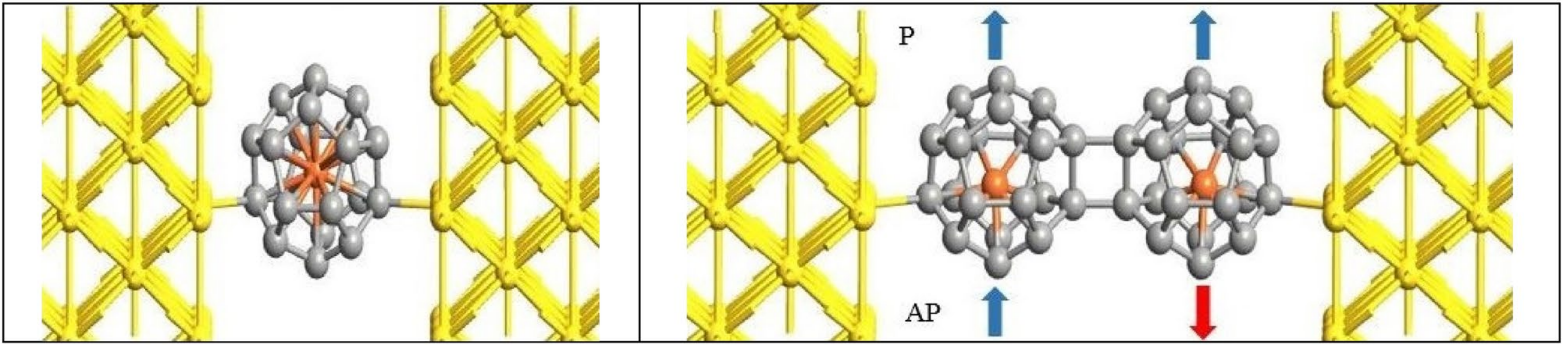
The geometrical structure of the Au-Fe@C_20_-Au (left) and Au-(Fe@C_20_)_2_-Au (right) systems. P and AP show the spin directions of the Fe atoms that are parallel and anti-parallel, respectively. The blue (red) arrow represents the spin-up (spin-down) direction for Fe atoms.

To form a dimer molecule, we use the [2 + 2] side-side mode which is the most stable geometry^4^. For the P configuration, the spin direction of the left and right Fe atoms are the same while for the AP configuration, the spin directions of the left and right Fe atoms are opposite. Geometry optimization process is done so that the net force on the atoms becomes less than 0.02 eV/Å. After optimization, the resultant molecules were placed between Au (100) electrodes and connected through a single contact to the electrodes. The structure of C_20_ fullerene in connection with the Au electrodes has been considered in various articles in recent years^14,22,41–50^. After the optimization of the considered structures, the obtained results in our calculations were very close to results calculated by other groups including^14,22,41,42^. In fact, the molecules are attached to the Au (100) electrodes with a separation of 2.185 Å^22,23^. Figure 2 shows the central scattering region of considered structure. We employed single zeta plus polarization (SZP) basis sets for Au atoms and double zeta plus polarization (DZP) basis set for other atoms. The transport direction is along z-axis, and the xy plane is perpendicular to the transport direction. The convergence test has been done to find the suitable k-point grid 3, 3 and 100 in the x, y and z directions by the Monkhorst Pack scheme^51^.

In the linear response regime, the electric and the heat currents in the spin channel, $\upsigma \left( { = \uparrow , \downarrow } \right)$, are respectively obtained by^52–54^,

1 $$\Delta {\text{I}}_{\upsigma } = {\text{e}}^{2} {\text{L}}_{0\upsigma } \Delta {\text{V}}_{\upsigma } + {\text{eL}}_{1\upsigma } ({{\Delta {\text{T}}} \mathord{\left/ {\vphantom {{\Delta {\text{T}}} {\text{T}}}} \right. \kern-\nulldelimiterspace} {\text{T}}}),$$

and

2 $$\Delta {\text{J}}_{\upsigma } = {\text{eL}}_{1\upsigma } \Delta {\text{V}}_{\upsigma } + {\text{L}}_{2\upsigma } ({{\Delta {\text{T}}} \mathord{\left/ {\vphantom {{\Delta {\text{T}}} {\text{T}}}} \right. \kern-\nulldelimiterspace} {\text{T}}}),$$

where ${\text{L}}_{{{\text{n}} = (0,1,2)\upsigma }} = \left( {{{ - 1} \mathord{\left/ {\vphantom {{ - 1} {\text{h}}}} \right. \kern-\nulldelimiterspace} {\text{h}}}} \right)\int {{\text{d}}\varepsilon_{{}} \left( {\varepsilon - \upmu } \right)^{{\text{n}}} \partial_{\varepsilon } {\text{f}}\left( \varepsilon \right){\text{T}}_{\upsigma } \left( \varepsilon \right)}$ is the Lorentz function and ${\text{f}}\left( \varepsilon \right) = 1/\left( {1 + \exp \left( {{{\varepsilon - \upmu } \mathord{\left/ {\vphantom {{\varepsilon - \upmu } {{\text{k}}_{{\text{B}}} {\text{T}}}}} \right. \kern-\nulldelimiterspace} {{\text{k}}_{{\text{B}}} {\text{T}}}}} \right)} \right)$ is the Fermi–Dirac distribution function of the electrode with chemical potential of $\upmu$ at T temperature. The spin-dependent transmission coefficient, ${\text{T}}_{\upsigma } \left( \varepsilon \right)$ obtained by TRANSIESTA code^55^ is given by:

3 $${\text{T}}_{\upsigma } (\varepsilon ) = {\text{Tr}}\left( {\Gamma_{{{\text{L}},\upsigma }} (\varepsilon )_{{}} {\text{G}}_{\upsigma }^{{\text{r}}} (\varepsilon )\Gamma_{{{\text{R}},\upsigma }} (\varepsilon ){\text{G}}_{\upsigma }^{{\text{a}}} (\varepsilon )} \right),$$

where $\Gamma_{{{\text{L}}\left( {\text{R}} \right)}} \left( \varepsilon \right)$ as the broadening matrix is equal to the anti-Hermitian part of the self-energy, $\Gamma_{{{\text{L}}\left( {\text{R}} \right)}} \left( \varepsilon \right) = {\text{i}}\left( {\upsigma_{{{\text{L}}\left( {\text{R}} \right)}} \left( \varepsilon \right) - \upsigma_{{{\text{L}}\left( {\text{R}} \right)}}^{\dag } \left( \varepsilon \right)} \right)$ and $\upsigma_{{{\text{L}}\left( {\text{R}} \right)}} \left( \varepsilon \right) = \uptau {\text{g}}_{{{\text{L}}\left( {\text{R}} \right)}} \left( \varepsilon \right)\uptau^{\dag }$ is the self-energy describing the contact between left (right) electrode and the molecule, which depends on the electrodes surface Green’s function, ${\text{g}}_{{{\text{L}}\left( {\text{R}} \right)}}$, and coupling matrix, $\uptau$. ${\text{G}}_{\upsigma }^{{{\text{r}}\left( {\text{a}} \right)}} \left( \varepsilon \right) = 1/\left( {\varepsilon {\text{S}} - {\text{H}} - \upsigma_{{\text{L}}} \left( \varepsilon \right) - \upsigma_{{\text{R}}} \left( \varepsilon \right)} \right)$ is the spin-dependent retarded (advanced) Green’s function of the central scattering region, where S and H are the overlap and the Hamiltonian matrix, respectively. The SP can be calculated as^56^

4 $${\text{SP}} = \left[ {({{{\text{T}}_{ \uparrow } (\varepsilon ) - {\text{T}}_{ \downarrow } (\varepsilon ))} \mathord{\left/ {\vphantom {{{\text{T}}_{ \uparrow } (\varepsilon ) - {\text{T}}_{ \downarrow } (\varepsilon ))} {({\text{T}}_{ \uparrow } (\varepsilon ) + {\text{T}}_{ \downarrow } (\varepsilon ))}}} \right. \kern-\nulldelimiterspace} {({\text{T}}_{ \uparrow } (\varepsilon ) + {\text{T}}_{ \downarrow } (\varepsilon ))}}} \right] \times 100,$$

where ${\text{T}}_{ \uparrow } \left( \varepsilon \right)$ and ${\text{T}}_{ \downarrow } \left( \varepsilon \right)$ are the transmission coefficients of the spin-up and spin-down channels, respectively. The positive (negative) amount of SP denotes a transport dominated by the spin-up (spin-down) channel. The SP value in the Fermi energy is known as spin-filter, which indicates the ability of system in filtering of carriers with a specific spin. The spin-dependent conductance (${\text{G}}_{\upsigma }$), thermopower (${\text{S}}_{\upsigma }$) and electron thermal conductance ($\upkappa_{{{\text{e}},\upsigma }}$) are calculated by^57^

5 $${\text{G}}_{\upsigma } = {\text{e}}^{2} {\text{L}}_{0\upsigma } ,$$

6 $${\text{S}}_{\upsigma } = - \lim_{{\Delta {\text{T}} \to 0}} \left. {\left( {{{\Delta {\text{V}}_{\upsigma } } \mathord{\left/ {\vphantom {{\Delta {\text{V}}_{\upsigma } } {\Delta {\text{T}}}}} \right. \kern-\nulldelimiterspace} {\Delta {\text{T}}}}} \right)} \right|_{{\Delta {\text{I}}_{\upsigma } = 0}} = \frac{1}{{{\text{eT}}}}\left( {{{{\text{L}}_{1\upsigma } } \mathord{\left/ {\vphantom {{{\text{L}}_{1\upsigma } } {{\text{L}}_{0\upsigma } }}} \right. \kern-\nulldelimiterspace} {{\text{L}}_{0\upsigma } }}} \right),$$

7 $$\upkappa_{{{\text{e}},\upsigma }} = \lim_{{\Delta {\text{T}} \to 0}} \left( {{{\Delta {\text{J}}_{\upsigma } } \mathord{\left/ {\vphantom {{\Delta {\text{J}}_{\upsigma } } {\Delta {\text{T}}}}} \right. \kern-\nulldelimiterspace} {\Delta {\text{T}}}}} \right) = \frac{1}{{\text{T}}}\sum\limits_{\upsigma } {\left[ {{\text{L}}_{2\upsigma } - \left( {{{{\text{L}}_{1\upsigma }^{2} } \mathord{\left/ {\vphantom {{{\text{L}}_{1\upsigma }^{2} } {{\text{L}}_{0\upsigma } }}} \right. \kern-\nulldelimiterspace} {{\text{L}}_{0\upsigma } }}} \right)} \right].}$$

The charge (spin) conductance and charge (spin) thermopower are ${\text{G}}_{{{\text{c}}\left( {\text{s}} \right)}} = \left( {{\text{G}}_{ \uparrow } \pm {\text{G}}_{ \downarrow } } \right)$ and ${\text{S}}_{{{\text{c}}\left( {\text{s}} \right)}} = \left( {{\text{S}}_{ \uparrow } \pm {\text{S}}_{ \downarrow } } \right)/2$, respectively. Finally, the charge (spin) figure of merit is defined as^58^,

8 $${\text{Z}}_{{{\text{c}}\left( {\text{s}} \right)}} {\text{T}} = \left( {{{\left( {{\text{S}}_{{{\text{c}}\left( {\text{s}} \right)}}^{2} {\text{G}}_{{{\text{c}}\left( {\text{s}} \right)}} } \right)} \mathord{\left/ {\vphantom {{\left( {{\text{S}}_{{{\text{c}}\left( {\text{s}} \right)}}^{2} {\text{G}}_{{{\text{c}}\left( {\text{s}} \right)}} } \right)} {\left( {\upkappa_{{{\text{e}}, \uparrow }} + \upkappa_{{{\text{e}}, \downarrow }} } \right)}}} \right. \kern-\nulldelimiterspace} {\left( {\upkappa_{{{\text{e}}, \uparrow }} + \upkappa_{{{\text{e}}, \downarrow }} } \right)}}} \right){\text{T}}.$$

## Results and discussion

**Figure 3:**
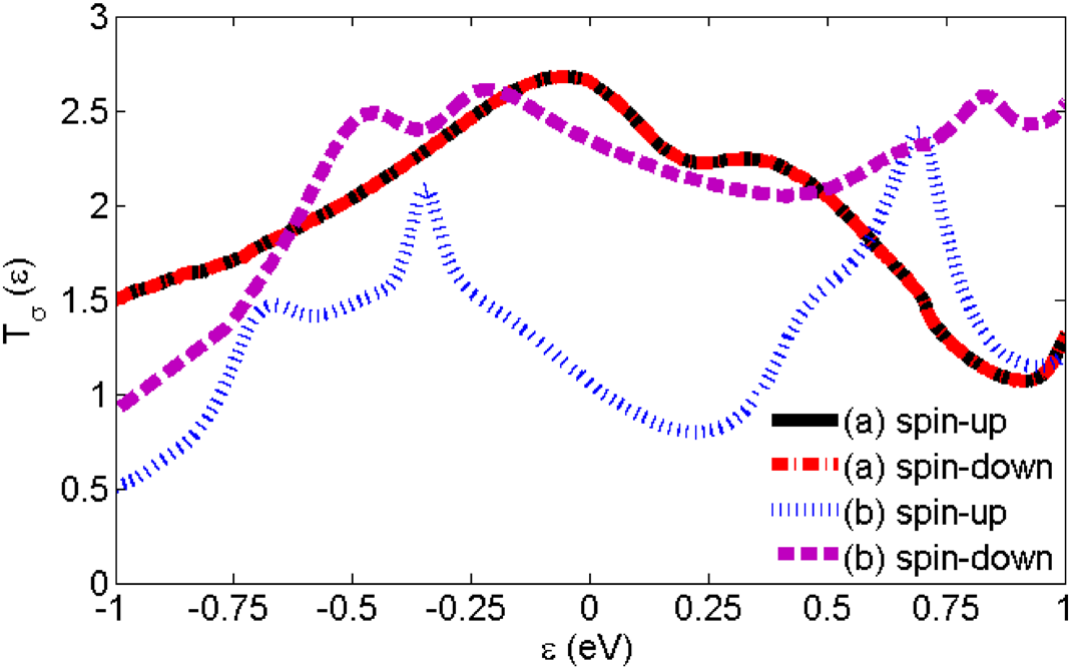
Spin-dependent transmission coefficient versus energy for (**a**) Au-C_20_-Au and (**b**) Au-Fe@C_20_-Au systems.

Figure 3 shows the spin-dependent transmission coefficient versus energy for two Au-C_20_-Au and Au-Fe@C_20_-Au systems. There is no clear difference between the spin-up and spin-down transmission peaks in Au-C_20_-Au system. However, the presence of Fe atom in the Au-Fe@C_20_-Au system causes a big difference in the spin-up and spin-down transmission peaks. By comparing the transmission of the two systems we clearly see a substantial decrease in the vicinity of Fermi level (E = 0 eV) while adding Fe atom. Obviously, the spin-up transmission of the Au-Fe@C_20_-Au system around the Fermi level has decreased substantially and it causes the spin-up and spin-down transmission to be distinguished easily. The transmission is directly related to the electronic structures and the transport properties of a system and having a larger transmission coefficient near the Fermi level means the strong transport capacity.

To have a clear understanding of the issue, we calculate the eigenvalues and eigenstates of transmissions in the Fermi level. Because in this level the transmission peaks are significantly different. The transmission can be obtained by sum of all the eigenvalues of the transmission matrix in a particular energy. The transmission eigenvalues are obtained by diagonalizing the transmission matrix^55^. On the other hand, the number of eigenvalues is an indicative of the number of channels through which the electrons pass by molecule and the strength of each channel can be determined by its eigenvalues. Transmission eigenvalues for Au-C_20_-Au and Au-Fe@C_20_-Au systems in E = 0 eV are listed in Table 2.

**Table 2:** Transmission eigenvalues for Au-C_20_-Au and Au-Fe@C_20_-Au systems in E = 0 eV.

Device	Spin-up	Spin-down
Au-C_20_-Au	0.95, 0.90, 0.76 and 0.21	0.95, 0.90, 0.76 and 0.21
Au-Fe@C_20_-Au	0.74, 0.18, 0.13 and 0.06	0.99, 0.91, 0.53 and 0.02

**Figure 4:**
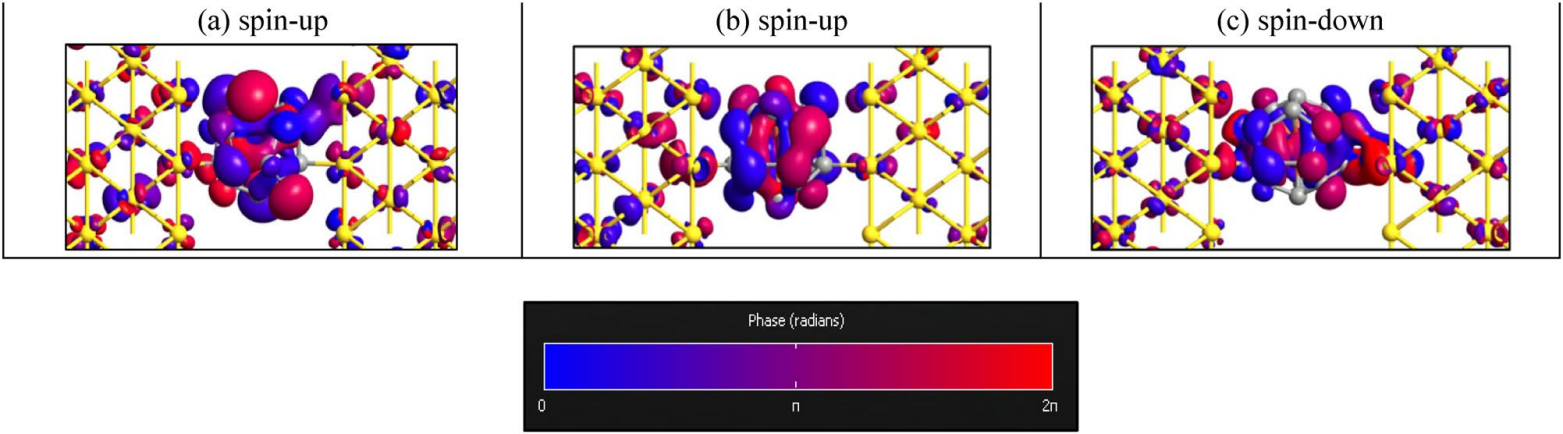
The phase diagram of the transmission eigenstates in zero energy for (**a**) Au-C_20_-Au and, (**b**) spin-up and (**c**) spin-down eigenstates of Au-Fe@C_20_-Au systems (Isovalue = 0.13).

Figure 4 shows the transmission eigenstate of the first transmission eigenvalue in zero energy for Au-C_20_-Au and Au-Fe@C_20_-Au systems. For Au-C_20_-Au system in Fermi level with the largest transmission peak values, we have calculated the transmission eigenvalues. The corresponding results are demonstrated in Fig. 4a. In zero energy, it is obviously visible that the spin-up transmission eigenstate (see Fig. 4a) is delocalized throughout the central regions from the left to the right and it causes a strong transmission in a particular energy. Both the spin-up and spin-down transmission eigenstates are equal, so we have just shown spin-up transmission eigenstate. Nevertheless, the transmission eigenstates of the spin-up carriers (see Fig. 4b) in Au-Fe@C_20_-Au system are localized which indicates that the transmission channels are suppressed, and as a result their transmission values are small at the desired energy. However, the transmission eigenstates of the spin-down carriers (see Fig. 4c) are delocalized which leads to the bigger transmission values at the desired energy.

**Figure 5:**
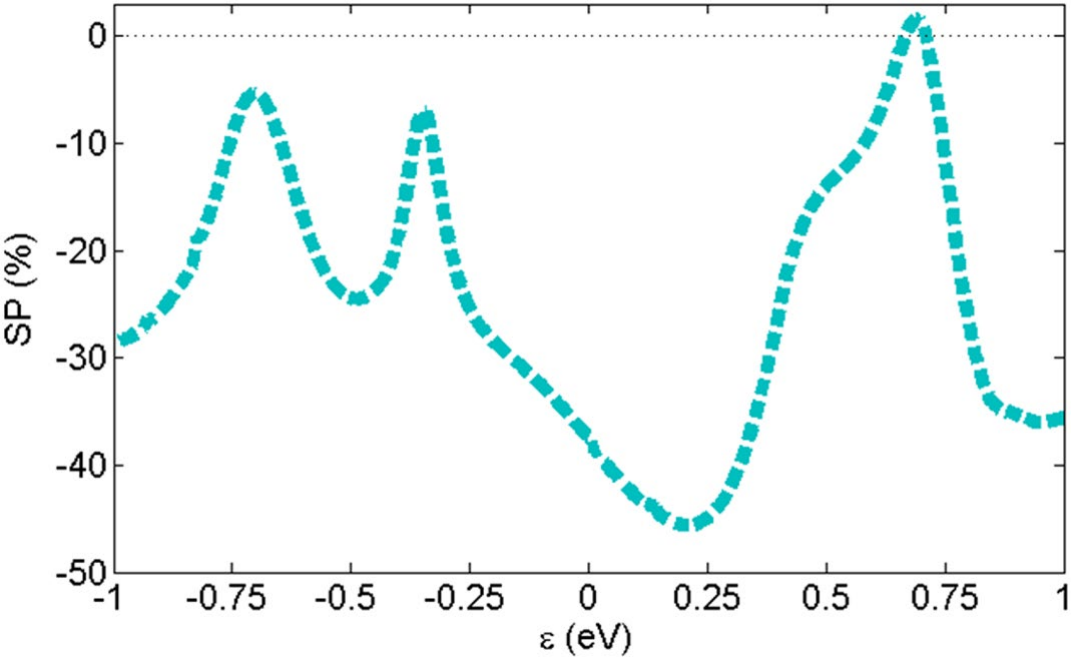
Spin polarization versus energy for Au-Fe@C_20_-Au system.

All the spin-up and spin-down transmission peaks in the Au-C_20_-Au system have the same values so it does not have SP. As we mentioned before, the value of spin-up and spin-down transmission peaks for the Au-Fe@C_20_-Au system are different and as a consequence leads to SP. The plot of SP versus energy has been shown in Fig. 5 for Au-Fe@C_20_-Au. The SP values are negative in all energies except those falls into the range of 0.67 until 0.71 eV where the spin-up transmission is higher than the spin-down transmission. Although the highest SP value in Au-Fe@C_20_-Au system is − 45% at energy 0.2 eV, in Fermi energy spin-filter efficiency (SFE) is equal to − 37%. This amount of SFE has been obtained in other work including^23^. Though these SP values in the Fermi energy make molecules promising structure in spintronics, this value is low in comparison with the spin-filters obtained in other works^21,22,59^. On the other hand, it is possible to achieve a higher SFE by increasing the length of the molecule. In the following, we aim to use dimer molecules to increase the SFE, which is in agreement with previous studies^60^.

**Figure 6:**
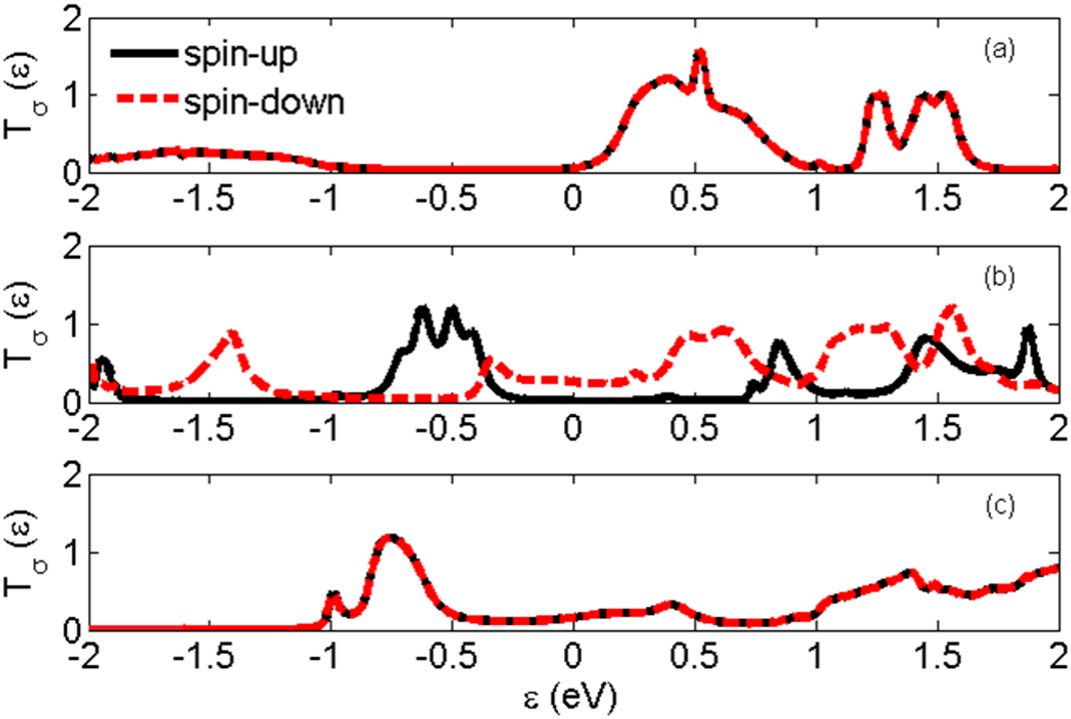
The spin-dependent transmission coefficient versus energy for (**a**) Au-(C_20_)_2_-Au, (**b**) Au-(Fe@C_20_)_2_-Au-P and (**c**) Au-(Fe@C_20_)_2_-Au-AP systems.

**Figure 7:**
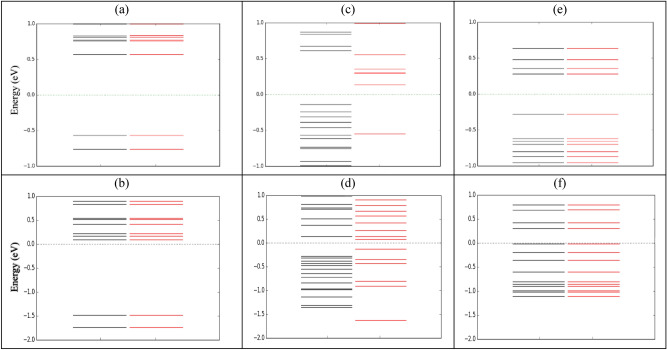
The molecular energy spectrum for (**a**) (C_20_)_2_, (**b**) Au-(C_20_)_2_-Au, (**c**) (Fe@C_20_)_2_-P, (**d**) Au-(Fe@C_20_)_2_-Au-P, (**e**) (Fe@C_20_)_2_-AP and (**f**) Au-(Fe@C_20_)_2_-Au-AP systems. The black and red lines represent the spin-up and spin-down, respectively. Fermi level is denoted by the horizontal dash line.

The spin-dependent transmission coefficients versus energy for two different systems; Au-(C_20_)_2_-Au and Au-(Fe@C_20_)_2_-Au have been shown in Fig. 6. The transmission of the Au-(C_20_)_2_-Au system decreases considerably in the regions of negative energy (energies lower than Fermi level) so that it becomes very close to zero. To clarify the results, we have plotted the molecular energy spectrum of the molecules without the electrodes and in the presence of the electrodes are presented in Fig. 7. It is observed that the number of energy levels is very small and therefore the number and the height of transmission peaks dramatically decrease in the regions of negative energy. The maximum transmission value occurs at energy 0.52 eV and the maximum charge conductance occurs in this energy (see Fig. 10a). The transmission reduction in regions of negative energy affects significantly both the electric and thermal conductance properties.

Figure 2 shows two possible spin configurations of Au-(Fe@C_20_)_2_-Au systems; P and AP which the spin orientations of Fe atoms are parallel and anti-parallel, respectively. An increase in the transmission around the Fermi levels and particularly in regions of negative energy is shown in Fig. 6. This is due to the presence of Fe atoms in the system which creates more energy levels especially in the regions of negative energy (see Fig. 7). Considering the P configuration, the transmission around the Fermi energy for spin-down is higher than that of for the spin-up and has also a broader peak. According to Fig. 7d, the lower distance between the energy levels in the spin-down case around the Fermi energy causes further transitions of electrons between the energy levels. Actually the transmission gap is smaller in the case of the spin-down in comparison with the spin-up. The densely compacted energy levels in the spin-up case causes to the significant enhancement of the transmission peaks in the interval [− 0.4 until − 0.7 eV]. For the AP configuration, similar to Au-(C_20_)_2_-Au system, the transmissions of spin-up and spin-down coincide. Due to the lack of symmetry of spins, this system is non-magnetic. Also, the same transmission peak values are essentially due to the same molecular energy levels. As we see in Fig. 7f, there is no molecular energy in the energy range − 1.1 to − 2 eV of the AP configuration. This is why we cannot see any transmission to occur in this specific energy range. Regarding Fig. 6 and comparing the transmissions around the Fermi energy, it is observed that the higher transmission belongs to spin-down of P configuration. According to Fig. 7d, gaps are smaller in this case and therefore higher amount of transition happens between the energy levels of electrode-molecule-electrode. By comparing the calculated transmission values (Figs. 3 and 6), it is clear that the transmission decreases with increasing the length of molecule^61^. Apparently, the longer the length of the device is, the lower the transmission will be. Although, in the dimer systems the enhancement of the length of the molecule has caused a decrease in the transmission but as we see in Fig. 9, increase of the molecule length causes a substantial increase in the spin-filter.

**Figure 8:**
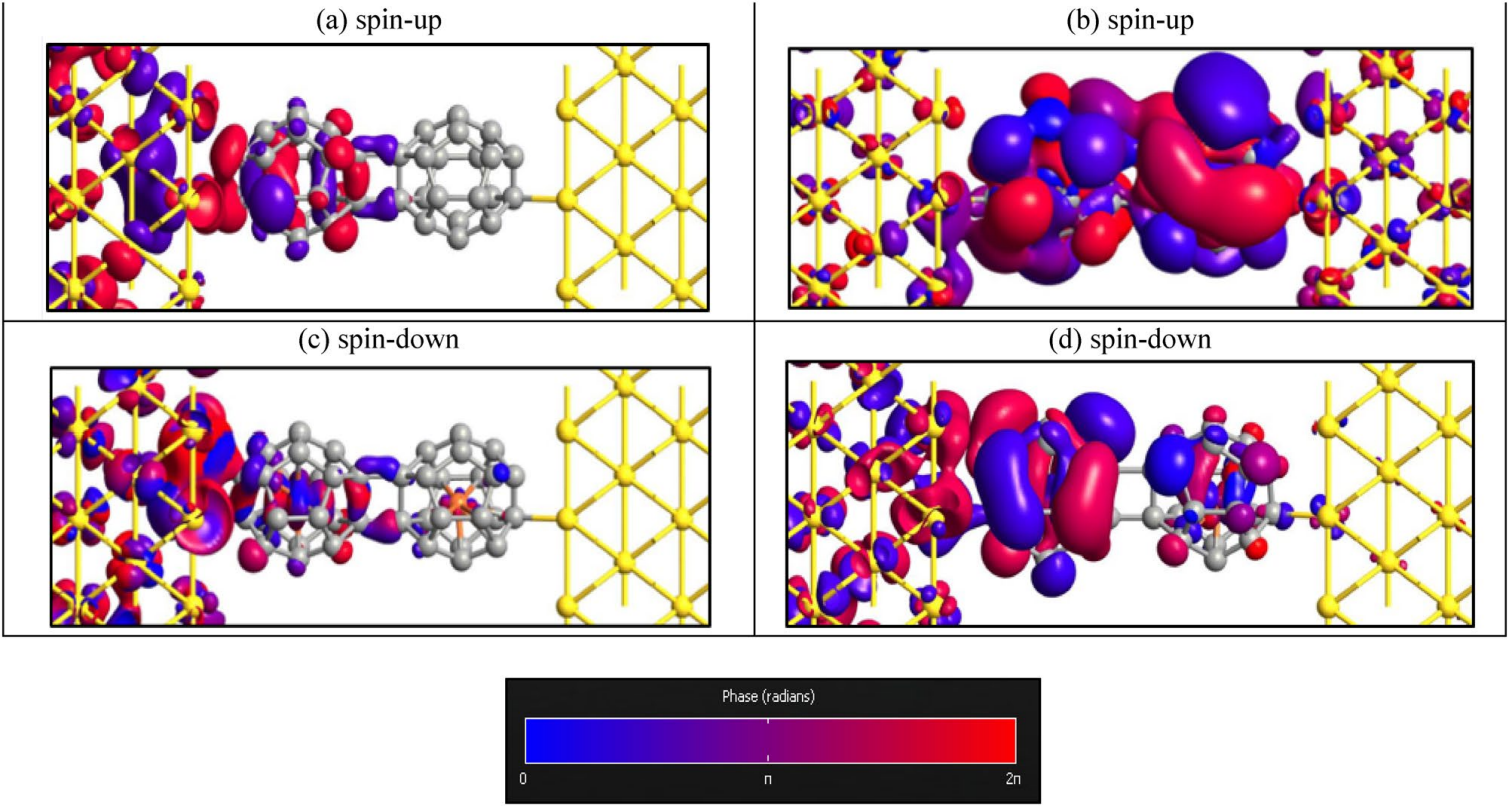
The phase diagrams of the transmission eigenstates in E = − 0.5 eV for (**a**) Au-(C_20_)_2_-Au, (**b**) spin-up (**c**) spin-down eigensates of Au-(Fe@C_20_)_2_-Au-P and (**d**) Au-(Fe@C_20_)_2_-Au-AP systems (Isovalue = 0.13).

As we mentioned before, it is obviously observed that the presence of Fe atoms in the dimer systems causes considerable peaks in the transmission especially in the regions of negative energy. On the other hand, the highest difference between transmission coefficients of spin-up and spin-down carriers is observed at energy points − 0.5 and − 0.62. So we have shown the eigenvalues and eigenstates of transmission for dimer systems in E = − 0.5 eV. For the Au-(Fe@C_20_)_2_-Au system in P configuration, among all the existent transport channels only two first channels for the spin-up with transmission eigenvalues of 0.98 and 0.22 have a main contribution in transferring electrons. However, in the case of AP, only one channel for the spin-up and spin-down has the main contribution for transport with transmission eigenvalue of 0.21 and the other eigenvalues are so small that can be negligible. For the Au-(C_20_)_2_-Au system and the spin-down of the P configuration of Au-(Fe@C_20_)_2_-Au, the eigenvalues are so small that the transmission becomes zero in E = − 0.5 eV. We found that the maximum eigenvalue belongs to the spin-up of the P configuration and the total summation of all the eigenvalues of transmission becomes larger than the others. So, the spin-up transmission in the P configuration in E = − 0.5 eV becomes larger than the others. Obviously, the wave function of the incoming electrons from the left electrode can reach the right electrode. The transmission eigenstates are related to a scattering state that comes from the left electrode and proceeds toward the right electrode. Therefore, the transmission eigenstates generally have comparatively large amplitude on the left side of the central region. In Fig. 8a, the amplitude of the eigenstates almost disappeared in the right side of the scattering region. By comparing Figs. 4a and 8a, we see that the transmission eigenstates are changed from delocalized to localized, which displays that the carriers transport is inhibited by the increase of the molecule length. In P configuration, while the addition of Fe atoms in the system causes to the appearance of the amplitude of the eigenstates in the right side of the scattering region for spin-up carriers, the amplitude of the eigenstates for the spin-down carrier is vanished. According to Fig. 8b,c, the spin-up transmission eigenstates in P configuration are delocalized while the spin-down ones are localized. In AP configuration (Fig. 8d), the transmission eigenstate is localized but the presence of the amplitude of the eigenstates in the right side of the scattering region is slightly more than Au-(C_20_)_2_-Au system.

**Figure 9:**
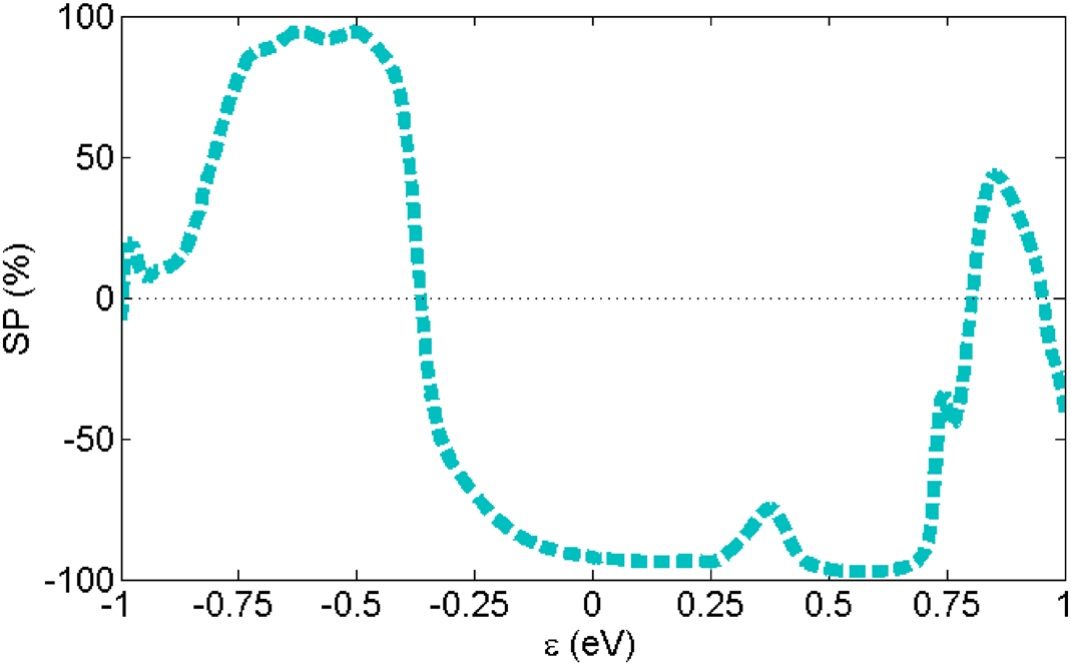
Spin polarization versus energy for Au-(Fe@C_20_)_2_-Au system in P configuration.

In Fig. 9, the SP curve versus energy is shown for Au-(Fe@C_20_)_2_-Au system in P configuration. In Fermi energy, SFE value for this system is equals to − 92%. The high SFE values close to the Fermi energy makes it a suitable system for spintronic applications. For the spintronic applications, the SFE of a system must be close to 100% around the Fermi energy, which is in agreement with references^22,59,62^. So, the longer molecular junctions are suitable for designing spin-filtering devices.

**Figure 10:**
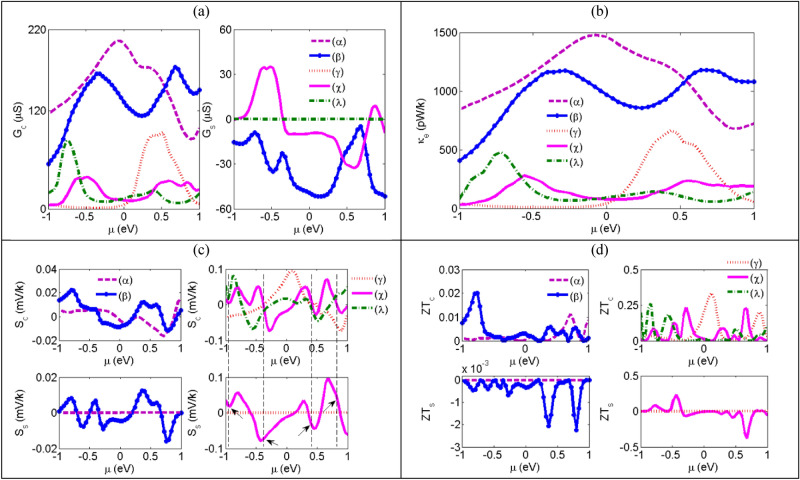
(**a**) the charge (left) and spin (right) conductance, (**b**) the electron thermal conductance, (**c**) the charge (first row) and spin (second row) thermopower and (**d**) the charge (first row) and spin (second row) figure of merit versus chemical potential of electrodes for (α) Au-C_20_-Au, (β) Au-Fe@C_20_-Au, (γ) Au-(C_20_)_2_-Au, (χ) Au-(Fe@C_20_)_2_-Au-P and (λ) Au-(Fe@C_20_)_2_-Au-AP systems at T = 300 K.

In the final section, we have calculated the thermoelectric coefficients of the considered systems versus the electrodes chemical potential at room temperature for both the monomer and dimer systems in Fig. 10. According to Fig. 10a, we see the charge conductance values are positive for all the systems. As we know, the charge conductance is the sum of spin-up and spin-down conductances and according to Eq. (5), these conductances values are related to L_0_ which is in turn obtained by the production of the transmission and the derivation of Fermi function. Since this production is a positive quantity we conclude that both the spin-up and spin-down conductance are positive. So, positive charge conductance is a consequence of positive spin-up and spin-down conductance. As we mentioned before, in Au-(C_20_)_2_-Au system, the charge conductance decreases substantially in the regions of negative energy and it reaches to a maximum value at E = 0.52 eV. The charge conductance directly related to the transmission. So, in those energies which the transmission reaches its minimum or maximum, the charge conductance shows the same behavior. By comparing the charge conductance of systems, it is obvious that similar to the transmissions, the charge conductance is decreased by the increment of the molecule length. Results show that unlike the charge conductance, the spin conductance shows different behaviors and it gains positive, negative or zero values. Among the considered systems, only the pure and AP systems spin conductance is zero. Because the spin-up and spin-down conductances are the same. Similar to the charge conductance, the electron thermal conductance decreases with increasing the length of molecule and the peaks have the same behavior (see Fig. 10b). Because at low temperature, the charge conductance is linearly proportional to electron thermal conductance^63^. The lack of transmission causes the charge conductance and electron thermal conductance peaks at only in positive chemical potentials and therefore, the transport in negative chemical potential decreases dramatically for Au-(C_20_)_2_-Au system in negative energies. Therefore, the negative chemical potentials for this system can be essentially considered as electric and thermal filters. In the following, we examined the charge and spin thermopower of considered systems versus the electrodes chemical potential in Fig. 10c. When the thermopower of spin-up becomes equal to that of spin-down, then the spin thermopower, which can be described as the difference between the spin-up and spin-down thermopower, becomes zero. This is why the spin thermopower of Au-C_20_-Au, Au-(C_20_)_2_-Au systems and the AP configuration of Au-(Fe@C_20_)_2_-Au become zero. When the charge thermopower becomes zero and the spin thermopower is non-zero (the vertical dashed lines in the Fig. 10c), a spin-dependent Seebeck effect happens^64,65^. In this case, the thermopower of spin-up and spin-down have the same magnitude but with different signs. We marked such points only for dimer systems because their non-zero values can be clearly seen. When the charge thermopower is zero, electrons and holes as the charge carriers have the same contribution in the transport. To have a non-zero amount for charge thermopower, it is necessary to destroy the symmetry between electrons and holes and so having an asymmetric conductance around the specific chemical potential. As we see in Fig. 10c, the spin and charge thermopower have positive, negative and zero values. When the charge thermopower is positive (negative) then the holes (electrons) are the main carriers in the transport process and the Fermi energy is also located near the HOMO (LUMO). The thermopower generally increase as the length of the molecule increases. There are also significant differences between the spin thermopower of P and AP configurations. The spin thermopower in the AP configuration is zero while that for P configuration is non-zero and owns a non-negligible value. The electron figure of merit is shown in Fig. 10d with neglecting phonon thermal conductance. According to the ZT formula, if we consider phonon thermal conductance, $\upkappa_{{{\text{ph}}}}$, the figure of merit is decreased while effect of phonons is important at high temperature. On the other hand, in the case of connecting a molecule to the electrode, the phonon thermal conductivity is very small and it can be ignored^66–70^. Figure 10d indicates that for the Au-Fe@C_20_-Au system the figure of merit around the Fermi level is bigger than that of for Au-C_20_-Au system. The bigger charge figure of merit for such a system is related to the higher effect of charge thermopower enhancement and also the electron thermal conductance reduction compares to the charge conductance decrease. The bigger spin figure of merit is related to enhance in both the spin thermopower and the spin conductance of the system and also to a decrease in its electron thermal conductance. The charge figure of merit of the Au-(C_20_)_2_-Au system has a bigger value in the positive chemical potential while it has dominant values for both P and AP configurations of Au-(Fe@C_20_)_2_-Au in negative chemical potential. Actually, in positive regions the effect of increasing the charge conductance and charge thermopower is considerably higher than the effect of increasing electron thermal conductance. This causes increasing the charge figure of merit in the Au-(C_20_)_2_-Au system. According to above explanations, the decrease of charge figure of merit in negative regions for this system is absolutely reasonable. In Fig. 10d, similar to thermopower, the figure of merit values is also increased by the increment of the molecule length. Equation (8) shows the proportionality of figure of merit to the square of thermopower. Therefore, the dimer systems own larger values of the figure of merit compare to the monomer ones. We can mark Au-(Fe@C_20_)_2_-Au-P system to be efficient to enhance the spin figure of merit and the Au-(C_20_)_2_-Au system to increase the charge figure of merit.

## Conclusions

We studied the impact of Fe atom in Au-Fe@C_20_-Au monomer and dimer systems in compared with Au-C_20_-Au system using density functional theory and non-equilibrium Green’s function formalism. In this study, we investigated various parameters, including spin-dependent transmission coefficient, transmission eigenvalue and eigenstate, molecular energy spectrum, spin polarization and finally their spin thermoelectric coefficients. The results show that the lack of transmission in negative energies for the Au-(C_20_)_2_-Au system makes charge conductance and electron thermal conductance peak only at positive chemical potentials, and the transfer in negative chemical potentials to be greatly decreased. This system can be considered as an electrical and also thermal filter in the negative chemical potential. On the other hand, the presence of Fe atoms in monomer and dimer systems has created spin distinct states. Considering the P and AP configurations, creates various modes for electron transmissions. The spin-filter in Au-Fe@C_20_-Au system about − 40% is achieved. While for Au-(Fe@C_20_)_2_-Au-P system, spin-filter is obtained − 92%. So, Au-(Fe@C_20_)_2_-Au-P system has many potentials for spintronic applications. We have found that Au-(Fe@C_20_)_2_-Au-P system to be highly efficient for increasing the spin figure of merit and Au-(C_20_)_2_-Au system for increasing the charge figure of merit.
